# Assessing Craving for Alcohol

**Published:** 1999

**Authors:** David J. Drobes, Suzanne E. Thomas

**Affiliations:** David J. Drobes, Ph.D., is an assistant professor and Suzanne E. Thomas, Ph.D., is a research instructor at the Center for Drug and Alcohol Programs, Medical University of South Carolina, Charleston, South Carolina

**Keywords:** AOD (alcohol and other drug) craving, assessment of variables and methods, specific AODU (alcohol and other drug use) measurement and test, AOD use behavior, psychophysiology, alcohol cue, temporal context, patient assessment, patient-treatment matching, disease severity, patient monitoring, self report, questionnaire, literature review

## Abstract

Craving for alcohol is common among alcohol-dependent people. Accordingly, measures to assess craving can play important roles in alcohol research as well as in alcoholism treatment. When developing or employing craving-assessment instruments, researchers and clinicians must consider numerous factors, such as the specific characteristics of craving to be evaluated, the instrument’s psychometric properties, and the timeframe over which craving is assessed. The measures most commonly used for assessing craving in clinical settings are single-item questionnaires, although several multi-item questionnaires also have been developed. Behavioral measures (e.g., amount of alcohol consumption or performance on cognitive tests) and psychophysiological measures (e.g., changes in salivation, respiration, or heart rate) are being used primarily in research settings. The assessment of craving can have numerous clinical benefits, such as helping the clinician to evaluate the severity of a patient’s alcohol dependence, to select appropriate treatment approaches, and to monitor changes throughout a patient’s treatment. The role of craving assessment in predicting treatment outcome, however, remains controversial.

The phenomenon of craving has received increasing attention in recent years from researchers and clinicians working with alcohol-related disorders. Scientists have developed several theoretical models of the development, neurobiology, and phenomenology of craving, some of which are described in other articles in this journal issue (e.g., see the articles in this issue by Anton, pp. 165–173, and by Tiffany, pp. 215–224). Despite increasing interest in this topic, little agreement exists on how best to conceptualize or measure craving. In addition, fundamental gaps in knowledge remain concerning the relationship between craving and actual drinking behavior.

As researchers and clinicians know from experience, craving is a common occurrence among alcohol-dependent people and a frequent topic of discussion in most alcoholism treatment and research settings. Accordingly, measures that accurately assess craving can play an important role in alcoholism research and treatment. This article reviews several critical issues associated with craving assessment and provides an overview of currently available methods for measuring alcohol craving. The article also provides some suggestions for assessing craving in routine clinical practice. Finally, it briefly discusses recent advances that may enhance the understanding and measurement of craving.

## Factors Affecting Craving Assessment

### Variables Assessed

The accuracy with which various instruments, or indices, measure craving for alcohol and other drugs (AODs) depends to a considerable extent on the type of variables the instruments evaluate. Early perspectives on alcohol craving focused on the subjective nature of craving. That is, craving was viewed as an experience that could only be assessed through the verbal report of the alcoholic. Consequently, the accuracy of most craving indices was limited by the ability and willingness of the individual alcoholic to accurately report his or her personal experience.

More recent conceptualizations of craving have fostered a broader perspective on the nature of craving and, consequently, on sources of data that could provide important information on craving. For instance, most researchers assume that craving among alcoholics is inexorably linked to alcohol consumption and to relapse to drinking in abstinent alcoholics. This perspective considers behaviors related to seeking or consuming alcohol as direct manifestations of craving. Some investigators, however, have challenged the assumption that alcohol-related behaviors directly result from craving on both theoretical and empirical grounds (see Tiffany 1990). Therefore, researchers must further determine for whom, when, and under what circumstances meaningful relationships may exist between craving and alcohol consumption.

Instruments that measure autonomic physiological activity^1^ (e.g., changes in heart rate, blood pressure, or sweat gland activity) in response to alcohol-related cues, such as the sight and smell of alcohol, also have received increased attention in studies of alcohol craving. Instruments that assess such autonomic responses to alcohol-related cues are particularly relevant to theories of craving that postulate a role for classical conditioning^2^ (e.g., Drobes et al. in press).

### Psychometric Issues

With the recent surge in interest in the theoretical and clinical importance of craving, researchers have attempted to develop ways to measure craving as well as ways to assess the utility of existing craving instruments. The utility of any craving assessment instrument depends on its psychometric properties; specifically, on whether the instrument reliably and validly measures craving.

The term “reliability” refers to the degree to which items on an instrument yield consistent results. Several types of reliability exist, not all of which are applicable to every type of assessment instrument. For example, test-retest reliability refers to the consistency of results when a subject is tested several times. Because craving is considered a relatively transient state that is expected to differ from one occasion to another, a high degree of test-retest reliability is not necessarily desirable for craving instruments. Other indices of reliability are more important to craving instruments—for example, whether consistent results are obtained with an instrument when several testers evaluate the same person (i.e., inter-rater reliability) and whether similar items on a test do not yield inconsistent or contradictory responses (i.e., internal consistency).

The term “validity” refers, in general, to the degree to which an instrument measures what it purports to measure. Because the empirical study of craving is a relatively new endeavor, and investigators consequently are still trying to understand exactly what craving is (and is not), assessing the validity of an instrument that proposes to measure craving is a difficult (and possibly premature) task. After more data have been generated and some agreement has been established on what constitutes craving, researchers can more confidently assess the validity of craving instruments.

As with reliability, several types of validity exist. Some of the most common measures of validity include construct validity, external validity, discriminant validity, and criterion-oriented validity. Construct validity refers to the degree to which results from an instrument reflect the underlying quality (e.g., craving) that the instrument is trying to measure. External validity describes the degree to which the results obtained with the instrument agree with results obtained with a different instrument with established validity. Discriminant validity indicates an instrument’s ability to accurately discriminate between populations with and without the quality of interest. For example, for alcohol craving, discriminant validity might be tested by whether the instrument discriminates alcoholics from nonalcoholics. Finally, criterion-oriented validity refers to how well scores on an instrument correlate with behaviors that are supposedly relevant to the quality being measured. For example, good criterion-oriented validity might be suggested if a craving instrument yields high scores for people who are willing to work for a drink in an experimental setting and low scores for people who are not willing to work for alcohol.

Most clinical and research assessments of craving have relied on brief measurement scales with unknown psychometric properties. In fact, the majority of investigators and clinicians have used instruments consisting of only a single question (i.e., single-item tests) to assess craving. Typically, these instruments ask questions such as, “How strong is your craving for alcohol?” or “How much do you crave an alcoholic beverage when you have gone without a drink for 1 to 2 days?” Because these indices consist of only one question, it is impossible to obtain an estimate of their internal consistency. Nonetheless, single items or brief scales that capture important aspects of the craving construct may demonstrate satisfactory inter-rater or test-retest reliability. Therefore, brief scales may provide especially useful tools where multiple craving assessments are desired and lengthier instruments would be impractical.

Alcohol cravings are generally thought to arise either from the desire to experience alcohol’s positive effects (i.e., positive reinforcement) or from the desire to avoid the negative effects of withholding alcohol, such as withdrawal symptoms (i.e., negative reinforcement). More recent models have suggested other important dimensions of craving, such as the desire and intention to consume alcohol, lack of control over alcohol use, and preoccupation with drinking-related thoughts and/or behavior. It is possible that certain aspects of craving characterize the experience of some alcoholics better than that of other alcoholics. Similarly, patients’ craving descriptions may change throughout the treatment process, necessitating measures that are sensitive to the dimensions of craving experienced at different time points or under differing circumstances. The development of indices that are sensitive to such differences may be helpful in the development of treatment plans based on the relative importance of various features of craving across individual patients and over time.

Unfortunately, the brevity of most available scales has not permitted researchers and clinicians to disentangle the multiple aspects of craving. Several newer multi-item instruments that take into account various dimensions of craving are discussed in the section “Methods of Assessing Alcohol Craving,” below. One should not assume, however, that each of these scales measures the same dimensions of craving or even that the same dimensional structure would be produced by the same instrument when administered to different samples of alcoholics (see Kranzler 1999).

### Timeframe of Assessment

Another critical aspect to consider when measuring craving is the timeframe of the assessment. Craving measures fall into two main categories with respect to their timeframe: (1) state measures, which focus on the patient’s current (“here-and-now”) craving status, and (2) global measures, which ask the patient to describe his or her general experience of craving over the course of 1 day, 1 week, 1 month, or an even longer time period.

The specific timeframe of the assessment instrument used should depend on the particular goals of the researcher or clinician administering the assessment. State measures are useful for assessing patients’ craving experiences at specific time points (e.g., during treatment sessions that expose the patients to alcohol or use other cognitive-behavioral approaches) and for determining changes in craving over time. Such immediate assessments of craving are particularly important in experimental settings, because they help researchers to better understand the neurological, biochemical, psychophysiological, cognitive, subjective, and emotional mechanisms involved in craving.

State measures may be less useful, however, in analyses assessing the relationship between craving and general alcohol use behavior. Studies typically have found only insignificant or weak relationships between the strength of subjective craving at a given time point and concurrent or subsequent alcohol use behavior. This lack of a correlation may result from the presence of multiple factors that may differentially influence subjective ratings and behavior. Examples of such factors include the patient’s internal state (e.g., mood or withdrawal symptoms), the presence of environmental cues related to past drinking, the perceived availability of alcohol, and the patient’s current motivation to consume alcohol.

In contrast to state measures of craving, global craving assessments tend to show a stronger relationship with actual alcohol use. One potential explanation for this stronger correlation is that alcohol-dependent people may be able to resist drinking during brief instances of craving, but cravings that occur frequently may have a cumulative impact on drinking. Therefore, instruments that ask patients to integrate their cravings over longer periods of time may offer a more reliable assessment of the extent to which cravings have caused distress or interference for the patient. This may, in turn, bear a closer connection to whether the person will engage in drinking behavior.

## Methods of Assessing Alcohol Craving

Assessment tools for craving generally fall into two broad categories: (1) self-report instruments and (2) behavioral and psychophysiological measures. Self-report instruments typically are questionnaires that can be either filled out by the patients themselves or administered by clinicians. These instruments are frequently used in both research and clinical settings. Behavioral and psychophysiological measures are primarily used in experimental settings.

### Self-Report Instruments

Self-report is the most frequently used method to obtain information about craving. In everyday clinical practice, therapists usually administer single-item instruments on which the patient reports his or her level of subjective craving. These instruments include questions such as “How strong is your craving for alcohol?” or “How strong is your urge to drink?” The therapist also provides anchor statements, such as “not at all” and “the most I’ve ever felt.” The patient then rates his or her craving either by selecting the most appropriate choice on a 7- or 10-point scale (i.e., a Likert-type scale) or by indicating a vertical mark along a line that connects the two anchor statements (i.e., a visual analog scale [VAS]). With VAS scales, the distance between the “no-craving” end of the line and the patient’s mark serves as the index of craving. For both types of assessment, the patient may be asked to rate his or her current level of craving or average level of craving over a longer time period.

Brief Likert-type and VAS scales provide a straightforward and time-effective approach to assessing a patient’s level of subjective craving. However, as noted earlier, these assessments are limited in their ability to provide information about the multiple elements that can define the craving experience (Tiffany 1992). Furthermore, researchers cannot determine the internal consistency of the instrument used. Because of these limitations, brief Likert-type or VAS ratings should be supplanted with multi-item instruments with desirable psychometric properties whenever possible. Several such instruments are described in the following paragraphs (also see table on p. 182).

#### The Yale-Brown Obsessive Compulsive Scale for Heavy Drinking (Y–BOCS–hd)

This scale, which was adapted from the Yale-Brown Obsessive Compulsive Scale (Y–BOCS) (Goodman et al. 1989*a*,*b*), was the first multi-item instrument developed and validated for the specific purpose of measuring craving for alcohol (Modell et al. 1992*a*). The Y–BOCS–hd conceptualizes alcohol craving as obsessions and compulsions relating to alcohol consumption. The term “obsession” refers here to the frequency and intrusive nature of thoughts about drinking, especially after 1 or 2 days of abstinence, whereas the term “compulsion” refers to the loss of control over drinking.

**Table:** Summary of Questionnaires for Assessing Alcohol Craving

Instrument	Source Reference (Supporting References)	Description	Administration	Psychometric Properties Reported
Yale-Brown Obsessive Compulsive Scale for heavy drinkers (Y–BOCS–hd)	Modell et al. 1992*a* (Goodman et al. 1989*a*)	Ten items in a structured interview; subscale scores for alcohol obsessions and compulsive behaviors related to alcohol can be obtained	Administered by a trained clinician; 15- to 30-min interview	Good psychometric properties reported on parent instrument (Y–BOCS); discriminant validity of Y–BOCS–hd supported by high sensitivity and specificity in correctly classifying alcoholics and normal controls
Obsessive Compulsive Drinking Scale (OCDS)	Anton et al. 1995 (Kranzler et al. 1999; Roberts et al. 1999)	Fourteen items; measure thoughts about alcohol during nondrinking period; can be scored for multiple subscales and factors	Self-administered questionnaire; requires 5 to 10 min to complete	High internal consistency (0.86); moderate to high internal consistency for individual subscales and factors (0.71–0.85); concurrent validity evidenced by strong correlations with measures of alcohol dependence severity and Y–BOCS–hd; good predictive validity shown by relationship between subscale scores and midtreatment drinking
Alcohol Urge Questionnaire (AUQ)	Bohn et al. 1995	Unidimensional Likert-type scale with eight items to assess acute craving	Self-administered questionnaire; requires 1 min to complete	High internal consistency (0.91); high test-retest reliability (1-day interval, 0.82); constuct validity evidenced by strong correlations with measures of alcohol dependence severity and the OCDS
Alcohol Craving Questionnaire (ACQ)	Singleton et al. 1994 (Love et al. 1998)	General form contains 47 items and short form contains 12 items; both forms provide measure of acute craving and can be scored for multiple subscales or factors	Self-administered questionnaire; general form requires 10 min and short form less than 5 min to complete	Moderate to strong factor (0.75–0.97) and subscale (0.77–0.86) reliability

Min = Minute(s)

NOTE: Discriminant validity indicates an instrument’s ability to accurately discriminate between populations with and without the quality of interest (e.g., alcoholics and nonalcoholics). Construct validity refers to the degree to which results from an instrument reflect the underlying quality (e.g., craving) that the instrument is trying to measure. Concurrent and predictive validity are types of criterion-oriented validity; they both describe the degree to which the results obtained with an instrument agree with other devices that purport to measure the same characteristic. Concurrent validity examines the relationship between measures taken around the same time, and predictive validity evaluates the relationship between the instrument and another measure taken at a later time. The reliability of an instrument indicates the degree to which items on an instrument yield consistent results. Test-retest reliability refers to the consistency of results when a subject is tested on multiple occasions. Internal consistency reliability indicates that similar items on a test do not yield inconsistent or contradictory responses. Reliability estimates can range from 0 to 1.0, with 1.0 indicating the highest degree of reliability.

The Y–BOCS–hd consists of 10 questions, of which 5 comprise an obsessionality subscale and the remaining 5 comprise a compulsivity subscale. The instrument is administered in a structured clinical interview lasting approximately 15 to 30 minutes.

In a study assessing the validity and reliability of the Y–BOCS–hd, Modell and colleagues (1992*b*) administered the questionnaire to 62 alcoholics. The results were then compared with ratings from a single-item subjective craving measure (i.e., “How much do you crave an alcoholic beverage when you’ve gone without a drink for 1 to 2 days?”). The study found that for both the total score and the two subscale scores, a statistically significant, albeit moderate, correlation existed between the ratings obtained with the two instruments.

#### The Obsessive Compulsive Drinking Scale (OCDS)

Anton and colleagues (1995) modified the Y–BOCS–hd to derive the Obsessive Compulsive Drinking Scale (OCDS), a 14-item questionnaire that the patient can self-administer and complete in about 5 minutes. Because the instrument is self-administered, each respondent is likely to interpret the questions similarly each time that he or she completes the questionnaire, thereby improving test-retest reliability and eliminating or reducing interviewer bias and differences in interpretation between interviewer and respondent. The OCDS is a global measure in which patients are asked to rate their craving over a period of 1 or 2 weeks (but no less than 1 day).

Like the Y–BOCS–hd, the OCDS contains an obsessive subscale, which consists of eight items, and a compulsive subscale, which consists of six items. However, the questions in the obsessive subscale on the OCDS relate to the occurrence of intrusive thoughts about alcohol at any time when the respondent is not drinking and thus encompass a more general timeframe than that specified in the Y–BOCS–hd. Patients respond to each item by selecting one of five statements that range from minimal to maximum endorsement of the item. The OCDS has demonstrated good test-retest reliability and high internal consistency; furthermore, its scores are strongly and significantly correlated with ratings obtained with the Y–BOCS–hd (Anton et al. 1995). The OCDS also has been shown to be a valuable tool for outcome assessment and for monitoring a patient’s progress during treatment (Anton et al. 1996).

#### The Alcohol Craving Questionnaire (ACQ)

The self-administered ACQ (Singleton et al. 1995) contains 47 items, each of which is scored on a 7-point Likert-type scale ranging from “strongly disagree” to “strongly agree.” Each item is related to one of five domains that are considered relevant to alcohol craving: (1) desire to drink alcohol, (2) intention to drink alcohol, (3) lack of control over the use of alcohol, (4) anticipation of positive effects from drinking (i.e., positive outcome expectancy), and (5) expectancy of relief from withdrawal or alcohol’s negative effects. The ACQ is a state measure providing an index of acute craving, because the questions relate to the degree to which the respondent is currently experiencing these urges. Initial validation work with the ACQ revealed four dimensions with moderate to high internal consistency. These factors were labeled emotionality, purposefulness, compulsivity, and expectancy (Singleton and Gorelick 1998). The ACQ also has been modified into a short form, the ACQ–SF, that contains the 12 items most strongly correlated with the total ACQ score.

#### The Alcohol Urge Questionnaire (AUQ)

The AUQ (Bohn et al. 1995) is an eight-item, self-administered state measure that assesses the patient’s urge for an alcoholic drink at the time the questionnaire is completed, thereby providing an index of acute craving. As with the ACQ, the items are scored along a seven-point Likert-type scale. The AUQ contains four items pertaining to the desire for a drink, two items regarding expectations of positive effects from drinking and two items relating to the inability to avoid drinking if alcohol were present. The AUQ has demonstrated good test-retest reliability, both after a 1-day and 1-week interval. Furthermore, the instrument has shown significant (but moderate) positive correlations with the patient’s alcohol-dependence severity and with scores on the OCDS, as well as a negative correlation with the length of time the patient has been abstinent (Bohn et al. 1995).

### Psychophysiological and Behavioral Measures

Researchers frequently use psychophysiological and behavioral indices in conjunction with self-report measures to elucidate the full range of responses that may relate to craving. A major advantage of measuring these additional response domains is that they provide more objective data than do self-reports and thus may be influenced less by various sources of bias. In addition, many craving theories make specific predictions regarding the physiological responses that should accompany craving reports and the relationships that should be observed across various indices of craving. The assessment of behavioral and psychophysiological variables can help support or disprove these predictions.

In laboratory settings, psychophysiological and behavioral responses are often measured within “cue reactivity” studies, which assume that the subject’s responses to alcohol-related stimuli can reflect craving (for more information on cue-reactivity studies, see the article in this issue by Litt and Cooney, pp. 174–178). Cues that have been used in studies of alcohol craving include the sight, smell, and taste of alcohol; pictures or videos of alcohol or alcohol-related scenarios, such as a barroom; the study participant’s belief that he or she will consume alcohol; and mental imagery of alcohol-related situations. Negative mood manipulations, which typically involve the participant imagining being in an unpleasant situation or being presented with a stressor that will invoke such a mood, also have been used as a cue for alcohol craving in some studies. Unfortunately, these studies have rarely demonstrated close relationships among behavioral, psychophysiological, and subjective measures of craving. Alcoholics’ responses to craving-induction procedures appear to vary considerably as a result of numerous individual differences and situational factors. Nonetheless, cue-reactivity measures have provided valuable information about the correlates of craving. Significantly, some studies have shown that autonomic reactions to alcohol cue presentations, such as changes in skin conductance,^3^ can predict later relapse to drinking (e.g., Drummond and Glautier 1994).

#### Psychophysiological Measures

Several psychophysiological variables have been assessed in response to alcohol cues, including salivation, skin conductance, skin temperature, respiration, heart rate, and blood pressure. Carter and Tiffany (1999) recently analyzed the results of numerous studies (i.e., conducted a meta-analysis) of cue-reactivity effects on psychophysiological variables across a range of AODs. Consistent with the assumption that alcoholics exhibit increased urges and cravings for alcohol in response to alcohol cues, this analysis revealed that alcoholics across studies demonstrate significant increases in heart rate and skin conductance in response to alcohol cues. That is, most studies showed that alcoholics responded with greater autonomic responses to alcohol cues than to control cues. However, substantial variability existed in the sizes of these effects among the studies included in this analysis, and it is important to note that the effects were generally larger for self-reports of craving than for the psychophysiological signals. The fact that most of these studies included single-item ratings suggests that as alluded to earlier, these items may indeed reliably assess craving in the context of cue-reactivity studies.

One physiological measure that was not included in the meta-analysis by Carter and Tiffany (1999) was salivation. Previous reviews have suggested that salivation increases reliably in the presence of alcohol cues (Niaura et al. 1988; Rohsenow et al. 1990–1991). This finding was confirmed in another recent meta-analysis, which also indicated a moderate increase in salivation among alcoholics in response to alcohol cues relative to control cues (Tiffany et al. in press).

#### Behavioral Measures

The behavioral measure most closely related to the concept of alcohol craving is alcohol consumption itself. Some researchers consider alcohol seeking and drinking during or after treatment to reflect, at least in part, craving processes. Various direct measures of drinking behavior can serve as outcome variables during or after alcoholism treatment, including frequency of drinking, number of drinks per drinking occasion (i.e., drinking intensity), and latency to drink.^4^ More fine-grained behavioral analyses of drinking patterns also have been conducted.

Although researchers generally are reluctant to conduct studies involving alcohol consumption with alcohol-dependent subjects, craving studies performed in laboratory rather than treatment settings occasionally include direct measures of alcohol consumption (Drobes and Anton in press). For example, Davidson and colleagues (1999) recently demonstrated that treatment with the medication naltrexone^5^ for 1 week diminished overall alcohol consumption and drinking speed in a laboratory session among young (i.e., with a mean age of 22 years) heavy drinkers.

Some recent cue-reactivity studies have incorporated indirect behavioral measures in lieu of actual drinking behavior. For example, Tiffany (1990) proposed that craving involves mental processes requiring conscious cognitive efforts (i.e., nonautomatic cognitive processing) and therefore should interfere with simultaneous activities that also require conscious cognitive effort (for more information on this model, see the article in this issue by Tiffany, pp. 215–224). To evaluate this hypothesis, Sayette and colleagues (1994) randomly assigned 24 alcoholic men admitted for inpatient alcoholism treatment to hold either a glass of water or their preferred alcoholic beverage for 3 minutes and to sniff the contents at specified intervals. Simultaneously, the men were instructed to respond by pressing a button whenever they heard a computer-generated tone. The reaction time on this button-pressing task was considered an indirect marker of the cognitive efforts associated with craving. The study found that during the period when the subjects were asked to sniff the beverages, the men holding the alcoholic beverage exhibited significantly longer reaction times than the men holding a glass of water, suggesting that the men in the alcoholic beverage group experienced greater craving.

Another indirect behavioral measure that may potentially serve as an indicator of alcohol craving is choice cue-viewing time—that is, the length of time a person chooses to look at a stimulus. Within studies on motivation and emotion, choice cue-viewing time has been related to various subjective and physiological indicators of arousal (e.g., Lang et al. 1993). Several other novel behavioral measurement approaches are currently being developed in the alcohol-craving research arena, some of which borrow methodologies from research on other drugs and from the emotion and motivation research field.

## Clinical Utility of Craving Measurements

Most alcoholics report that cravings are a frequent and troublesome aspect of their addiction. Consequently, clinicians should monitor their patients’ cravings. Assessment and discussion of craving may improve rapport between clinicians and patients, thereby increasing the relevance of therapeutic interactions. In everyday clinical practice, self-report instruments will likely continue to be the most frequently employed type of craving measure, because behavioral and psychophysiological measures, despite their theoretical relevance, clearly extend beyond the resource capacities of most treatment settings.

Incorporating craving measurements into routine clinical practice can produce several potential benefits. First, craving-related measures appear to provide a good index of a patient’s overall level of alcohol dependence. Anton and Drobes (1998) reported that alcoholics scored increasingly higher on the OCDS as a function of the severity of their dependence and, consequently, level of treatment involvement. That is, OCDS scores increased in order from nonalcoholics, to non-treatment-seeking alcoholics (i.e., patients with a low level of dependence severity), to alcoholics receiving outpatient therapy, to alcoholics in inpatient treatment (i.e., patients with the greatest dependence severity).

Second, craving assessment can provide valuable information concerning a patient’s capacity to recognize and monitor internal states that may be related to his or her drinking. If a patient can be taught to recognize his or her specific level of craving, then that person may be more likely to use learned coping strategies at the appropriate time or to return for additional treatment sessions as craving-inducing situations arise during recovery.

Third, clinicians may find it useful to assess craving in order to recommend appropriate treatment alternatives. For instance, studies have suggested that alcoholics who report higher levels of craving benefit the most from the medication naltrexone as an adjunct to psychosocial treatment components (Jaffe et al. 1996).

Finally, because craving measures have been shown to predict alcohol consumption during active treatment (Anton et al. 1995), regular monitoring of craving throughout treatment may aid clinicians and patients in decisions regarding treatment intensity and duration. For example, patients who continue to experience high levels of craving (either constantly or in certain situations) despite compliance with their current treatment may require continued support, more intensive treatment, or possibly changes in their environment (e.g., social network and activities).

The relationship between craving and drinking outcome is still somewhat controversial in the alcohol field. Some studies have demonstrated that self-reported craving can predict treatment outcome or relapse, whereas other studies report that acute craving often does *not* lead to alcohol consumption in either active or relapsing alcoholics. One explanation for this discrepancy may involve the types of measures used to assess craving. It is possible that global measures of craving are more strongly related with drinking behavior than are state-oriented measures, because global measures involve the cognitive integration of craving-related experiences over a longer time period. As a result, such measures may more reliably incorporate aspects of craving that cannot be obtained from state measures of craving. In particular, global measures may be better suited to assess the overall anguish and distress experienced by an alcoholic, which may be related to the likelihood of relapse. Conversely, state-oriented measures only give a snapshot of an alcoholic’s immediate alcohol-related struggle. Whether drinking occurs at the time of the assessment, however, likely depends on several other factors.

Nevertheless, a pattern of high scores on state-oriented measures of craving, while probably unrelated to drinking at a particular time, may show a similar positive relationship with eventual drinking as do more global measures. Although this hypothesis needs to be tested empirically, its tenet is that clinicians need to regularly administer both state-oriented and global measures of craving in order to estimate both current and general levels of alcohol-related distress. Such an approach could help optimize clinical judgments regarding the probability of relapse and the most appropriate interventions to prevent relapse.

## Summary and Future Directions

The past decade has witnessed the development and increased availability of psychometrically sound and theoretically relevant self-report measures of craving. The measures described in this article are better able to capture the dynamic and multidimensional nature of craving across and within individual patients over time compared with traditional single-item assessments. These measures also can be used in a wide range of research and clinical settings according to the needs of the administrator. Additional work is necessary, however, to further refine and validate these measures. In particular, the generalizability of the available measures to various types of drinking populations must be evaluated, because different subpopulations of alcoholics (e.g., treatment-seeking, non-treatment-seeking, abstinent, and adolescent alcoholics) may require new and unique craving instruments. The currently available measures can serve as useful guides in the development and validation of such instruments.

Technological advances are likely to improve the multidimensional measurement and understanding of craving in the coming years. For example, researchers have begun to apply neuroimaging techniques, such as functional magnetic resonance imaging (fMRI) and positron emission tomography (PET), in studies of craving (for more information on these techniques, see the article in this issue by Hommer, pp. 187–196). These new tools will be critical for identifying the brain regions activated concurrent with alcohol craving, particularly when combined with other existing technologies for measuring craving. Improvements in existing cue-reactivity approaches also hold promise for advancing measurements of craving phenomena. For example, methodological advances within the general study of emotion and motivation could be adapted for alcohol-cue reactivity analyses in order to better elucidate the role of emotions in alcohol craving. Such investigations are particularly important in light of recent studies demonstrating a link between emotion, cue reactivity, and treatment outcome (Cooney et al. 1997; Litt et al. 1990).

## Footnotes

1 Autonomic physiological responses are those reactions that are not normally controlled consciously, such as heart rate and blood pressure.

2 Classical conditioning is the process by which an originally neutral stimulus (e.g., sight of a bar) can induce a predictable response (e.g., an increase in heart rate) if it is repeatedly paired with another stimulus which elicits that response (e.g., alcohol consumption).

3 The term “skin conductance” refers to the ability of the skin to conduct weak electrical currents. This ability varies depending on the amount of moisture on the skin and can therefore be used as a measure of sweat gland activity.

4 The term “latency to drink” in this context refers to the elapsed time from when alcohol is first made available to the point at which consumption begins.

5 Naltrexone is a medication that has been approved by the U.S. Food and Drug Administration for the treatment of alcoholism when used in conjunction with psychosocial therapy. Although the exact mechanism of action of this agent is unknown, one hypothesis is that naltrexone may reduce craving.
